# Jasmonic acid enhances thermotolerance in hybrid Pennisetum via activation of the α-linolenic acid metabolism pathway

**DOI:** 10.3389/fpls.2026.1756886

**Published:** 2026-05-26

**Authors:** Zhitong Chen, En Chen, Jinhong Zhang, Zhaoyang Ying

**Affiliations:** Institute of Resources, Environment and Soil Fertilizer, Fujian Academy of Agricultural Sciences/Fujian Engineering and Technology Research Center for Hilly Prataculture, Fuzhou, China

**Keywords:** gene expression regulation, heat stress, heat tolerance, jasmonic acid, Pennisetum americanum×p.purpureum, α-linolenic acid metabolism

## Abstract

In this study, we investigated the role of jasmonic acid (JA) in the heat tolerance of Pennisetum americanum×P.purpureum cv.Minmu^6^; high temperatures generally cause leaf yellowing, dehydration, and reductions in chlorophyll and water contents, indicating photosynthetic disruption, and RNA-seq revealed significant α-linolenic acid metabolism pathway enrichment, with key JA biosynthesis genes (*PLA2H3*, *LOX8*, *AOS1*, *AOS2*, *OPR*1, and *OPR11*) markedly upregulated and endogenous JA levels elevated. Exogenous JA application alleviated this heat damage, maintained higher chlorophyll and water contents, and enhanced JA-related gene expression; qPCR confirmed JA’s positive feedback regulation within this pathway, with *LOX8* and *OPR11* showing strong responsiveness. Overall, heat stress activates α-linolenic acid metabolism to promote JA accumulation, while exogenous JA amplifies this response, improving heat tolerance; these findings elucidate a JA-mediated regulatory mechanism of thermotolerance and provide a theoretical basis for enhancing heat resilience in forage crops.

## 1 Introduction

Pennisetum americanum×P.purpureum cv.Minmu^6^, a perennial grass species of hybrid Pennisetum with excellent environmental adaptability, favorable morphological traits, and high biomass accumulation, is widely used in landscaping, ecological restoration, and livestock production in warm regions worldwide (Xu et al., 2023). The species is characterized by its distinctive panicle inflorescences and tufted leaf clusters, making it a popular ornamental plant for landscape design. In addition, its well-developed root system and high biomass contribute significantly to soil and water conservation, as well as ecosystem recovery. Certain cultivars of hybrid Pennisetum also possess high nutritional value and good palatability, serving as a valuable forage resource that supports the sustainable development of animal husbandry (Teng et al., 2023).

With the ongoing global climate warming, the frequency of extreme high-temperature events has increased significantly, posing serious challenges to plant growth, development, and agricultural ecosystems (Sato et al., 2024). Elucidating the heat tolerance mechanisms of hybrid Pennisetum is, therefore, of great theoretical and practical importance for understanding its ecological adaptation strategies and for improving forage production and heat-tolerant ornamental plant breeding under high-temperature conditions. During long-term evolution, plants have developed complex signaling networks through which they cope with environmental stresses; among them, JA, a lipid-derived phytohormone, has been widely recognized as a key regulator involved in plant responses to both biotic and abiotic stresses (Wang et al., 2021; Muhammad et al., 2023).

The biosynthesis of JA begins with the α-linolenic acid located in chloroplast membranes; in this pathway, phospholipase A (PLA) first catalyzes the release of α-linolenic acid from membrane lipids. Subsequently, lipoxygenase (LOX), allene oxide synthase (AOS), and allene oxide cyclase (AOC) sequentially catalyze its conversion to 12-oxo-phytodienoic acid (12-OPDA), an intermediate which is then reduced by 12-oxophytodienoate reductase (OPR) and further processed through β-oxidation to generate jasmonic acid (JA). In planta, JA can be further conjugated with amino acids, most notably isoleucine, to form jasmonoyl-isoleucine (JA-Ile), which is recognized as the primary biologically active jasmonate perceived by the COI1–JAZ receptor complex. Therefore, the regulation of JA biosynthesis provides the essential hormonal pool required for the production of bioactive JA-Ile and subsequent activation of JA-dependent responses. The expression levels of genes encoding these key enzymes directly determine the efficiency of JA biosynthesis and indirectly influence downstream jasmonate signaling, representing crucial molecular nodes in the regulation of plant stress responses (Li et al., 2021; Chini et al., 2023).Previous studies have demonstrated that JA and its derivatives play pivotal roles in plant responses to various abiotic stresses, including heat, drought, salinity, and oxidative stress (Muhammad et al., 2023; Ma et al., 2024). JA enhances stress tolerance by regulating antioxidant enzyme systems, stabilizing membrane structures, controlling stomatal movement, and inducing defense-related gene expression (Su et al., 2021). However, the JA-mediated responses to heat stress vary considerably among plant species, depending on their intrinsic traits and stress intensity (Otálora et al., 2022; Wu et al., 2022). To date, the physiological roles and molecular regulatory mechanisms of JA in the heat adaptation of hybrid Pennisetum remain largely unexplored (Meiling et al., 2023).

In order to investigate the potential role of JA in the development of heat tolerance in hybrid Pennisetum, plants were subjected to high-temperature conditions and sprayed with different concentrations of exogenous JA. Changes in leaf coloration and physiological status were observed, and the expression levels of six key genes involved in the JA biosynthetic pathway (*PLA2H3*, *LOX8*, *AOS1*, *AOS2*, *OPR1*, and *OPR11*) were analyzed using quantitative real-time PCR (qPCR). By integrating phenotypic observations with molecular data, we conducted this study with the aim of elucidating the regulatory role and mode of action of JA in the heat response of hybrid Pennisetum, thereby providing a theoretical basis for understanding the molecular mechanisms underlying heat tolerance in forage grasses, as well as for potential JA-based applications in stress resistance improvement.

## 2 Materials and methods

### 2.1 Plant materials

The experimental material used in this study was *Pennisetum americanum×P.purpureum* cv.Minmu ^6^, one cultivar of hybrid Pennisetum. Healthy plants with uniform growth, free from pests and diseases, were selected as experimental subjects. The plants were cultivated in an artificial climate chamber using a mixed substrate of peat soil and perlite (3:1, v/v). The substrate was kept moderately moist under controlled environmental conditions, with a light/dark cycle of 12 h/12 h, temperatures of 28 °C/25 °C (day/night), a light intensity of approximately 6000 lx, and a relative humidity of 60–70%.

### 2.1.1 Temperature treatments

The plants were randomly divided into three treatment groups, each with three biological replicates. The treatments were set as follows: CK (control): L/D = 12/12 h, 28 °C/25 °C; MT (moderate heat stress): L/D = 12/12 h, 35 °C/25 °C; HT (severe heat stress): L/D = 12/12 h, 45 °C/25 °C.

#### 2.1.2 Temperatures and JA treatments

The plants were randomly divided into three treatment groups, and leaves were sprayed with JA solutions of different concentrations (0 μmol/L, 50 μmol/L, and 100 μmol/L). The exogenous JA was purchased from Sigma-Aldrich (St. Louis, MO, USA; Cat. No. J2500). To ensure proper dissolution, the JA stock solution was first dissolved in a small volume of absolute ethanol and subsequently diluted with distilled water containing 0.01% (v/v) Tween-20 to the final working concentrations. After spraying, the plants were kept in dark conditions for 12 hours to ensure adequate absorption of JA. Subsequently, the plants from each treatment were exposed to three temperature regimes: CK (control, 28 °C), MT (moderate heat stress, 35 °C), and HT (severe heat stress, 45 °C). Each treatment included three biological replicates.

All treatments were conducted in a temperature-controlled artificial climate chamber. After 7 days of treatment, five plants were randomly selected from each biological replicate, and the third fully expanded leaf (counted from the apex) of each plant was collected and pooled as one composite sample. The samples were immediately frozen in liquid nitrogen and stored at −80 °C until further analysis, including RNA extraction and biochemical assays.

### 2.2 Physiological measurements

In order to evaluate the physiological responses of hybrid Pennisetum under different temperature treatments, the chlorophyll content and relative water content (RWC) of leaves were determined. Samples were collected from the third fully expanded leaf (counted from the apex) at the end of the light period. Each treatment included three biological replicates, and each replicate consisted of a mixture of equivalent leaves from five plants. Three technical replicates were performed for each sample.

#### 2.2.1 Determination of chlorophyll content

Chlorophyll content was measured using a Plant Chlorophyll Content Assay Kit (AK0302-50T/48S; Shanghai Sunlong Biotech Co., Ltd., China) following the manufacturer’s instructions; this kit is based on visible spectrophotometry and allows simultaneous quantification of chlorophyll a, chlorophyll b, and total chlorophyll content.

Approximately 0.10 g of fresh leaf tissue was homogenized with the extraction buffer provided in the kit. The homogenate was kept in the dark at room temperature for 15–30 min and centrifuged at 12,000 × g for 5 min at 4 °C. The supernatant’s absorbance was measured at 663 nm and 645 nm (A_663_, A_645_) using a spectrophotometer. Chlorophyll concentration (mg·L^-^¹) was calculated from absorbance or standard curves, and total chlorophyll content (mg·g^-^¹ FW) was determined using the following formula:

Chlorophyll content (mg\cdotpg^-^¹ FW)=1000×W(g)C (mg\cdotpL^-^¹)×V(mL).

where C is the chlorophyll concentration, V is the extraction volume (mL), and W is the fresh weight of the sample (g). All procedures were conducted in the dark to prevent pigment degradation, and three technical replicates were performed for each measurement.

#### 2.2.2 Determination of relative water content

RWC was determined using the classical fresh weight–turgid weight–dry weight (FW–TW–DW) method. Five of the third fully expanded leaves were randomly selected from each biological replicate. Fresh weight (FW) was measured (to the nearest 0.1 mg) after gently blotting surface moisture with filter paper. Samples were then immersed in distilled water and kept in the dark for 4 h (or stored at 4 °C for 4–6 h) to obtain the turgid weight (TW). Subsequently, the leaves were dried at 80 °C for 48 h (or until constant weight) to determine the dry weight (DW).

Relative water content was calculated using the following equation:

RWC (%)=(TW−DW/FW−DW)×100%.

Results are expressed as mean ± standard error (SE). Statistical significance was analyzed using one-way analysis of variance (ANOVA), followed by Duncan’s multiple range test (p < 0.05). Data visualization and statistical analyses were performed using GraphPad Prism 9 software (GraphPad Software, San Diego, CA, USA).

#### 2.2.3 Extraction and Quantification of JA, ALA, and JA-Ile

Fresh leaf samples (approximately 100 mg) collected after the 5-day temperature treatments were immediately frozen in liquid nitrogen and ground into a fine powder. The powder was homogenized in 1 mL of a pre-cooled extraction solvent consisting of methanol/water/acetic acid (80:19:1, v/v/v). Prior to extraction, known amounts of isotopically labeled internal standards, including d5-JA, d5-ALA (or an equivalent internal standard), and d6-JA-Ile, were added to each sample to ensure accurate quantification.

The homogenate was thoroughly vortexed and incubated at 4 °C for 12 h in the dark with continuous shaking. Subsequently, the samples were centrifuged at 12,000 × g for 15 min at 4 °C. The supernatant was collected and evaporated to dryness using a vacuum concentrator. The dried residue was reconstituted in 100 μL of 50% (v/v) methanol, vortexed, and centrifuged again to remove any insoluble particles. The final supernatant was filtered through a 0.22-μm membrane filter and transferred into autosampler vials for LC-MS/MS analysis.

Chromatographic separation was performed using an Agilent 1290 Infinity II liquid chromatography system coupled to a SCIEX QTRAP 6500+ mass spectrometer. Analytes were separated on a Waters ACQUITY UPLC BEH C18 column (2.1 mm × 100 mm, 1.7 μm) maintained at 40 °C. The mobile phases consisted of (A) 0.1% formic acid in water and (B) 0.1% formic acid in acetonitrile.

Mass spectrometry was operated with an electrospray ionization (ESI) source in negative ion mode, and quantification was performed using multiple reaction monitoring (MRM). The optimized MRM transitions (precursor ion → product ion) monitored were m/z 209.1 → 59.0 for JA, m/z 277.2 → 261.2 (or 233.2) for ALA, and m/z 322.2 → 130.1 (or 85.0) for JA-Ile. Transitions for the corresponding isotopic internal standards were monitored simultaneously.

The concentration of JA was calculated based on the ratio of the endogenous JA peak area to that of its internal standard, normalized to the fresh weight of the sample, and expressed as ng/g FW. The concentrations of ALA and JA-Ile were similarly determined using their respective peak area-to-internal standard ratios but were quantified based on the extraction volume, with the final results expressed as μg/L.

### 2.3 RNA extraction, full-length cDNA library construction, and structural analysis

Total RNA was extracted using a Polysaccharide Polyphenol Plant Total RNA Extraction Kit (Magen, China; Cat. No. R4150-02). RNA concentration and purity were determined with a NanoDrop ND-1000 spectrophotometer (Thermo Fisher Scientific, USA), and RNA integrity was assessed using an Agilent 2100 Bioanalyzer (Agilent Technologies, USA). Samples with purity (A260/A280 = 1.8–2.1) and integrity (RIN ≥ 7.0) meeting the required standards were used for subsequent library construction.

Equal amounts of RNA from different treatments were pooled and reverse-transcribed into full-length cDNA using the NEBNext Single Cell/Low Input cDNA Synthesis & Amplification Module (New England Biolabs, Ipswich, USA). After quality assessment, the cDNA libraries were sequenced on the PacBio Sequel II platform (Pacific Biosciences, Menlo Park, USA). Raw sequencing data were processed using SMRT Link software (v10.1) to generate circular consensus sequences (CCSs) with the following filtering parameters: full passes ≥ 3; average quality ≥ 0.9. Reads containing poly(A) tails and complete 5′ and 3′ primers were defined as full-length (FL) sequences. Primer and poly(A) tail sequences were removed using Lima v2.1.0, and sequence refinement and clustering were performed using the IsoSeq3 pipeline. High-quality consensus sequences (accuracy > 99%) were obtained through the ICE (Iterative Clustering and Error Correction) algorithm. The resulting FL sequences were used for subsequent structural and functional annotation analyses.

### 2.4 Transcriptome annotation and differential expression analysis

High-quality sequences were processed using CD-HIT to remove redundancy, and non-redundant representative transcripts were retained as reference sequences. Open reading frames (ORFs) were predicted using ANGEL software to extract complete coding sequences (CDSs) and their corresponding amino acid sequences. Functional annotation was performed with BLASTx (E-value ≤ 1e−5) against multiple databases: NR, Swiss-Prot, Pfam, Gene Ontology (GO), Kyoto Encyclopedia of Genes and Genomes (KEGG), and Eukaryotic Orthologous Groups (KOG). GO annotation was conducted using Blast2GO; and KEGG pathway annotation was obtained through the KAAS platform.

Clean reads from samples under different temperature treatments were aligned to the reference transcriptome using Bowtie2, and gene expression levels were calculated as fragments per kilobase of transcript per million mapped reads (FPKM) using RSEM. Differentially expressed genes (DEGs) were identified using DESeq2, with thresholds set at |log_2_FoldChange| ≥ 1 and a false discovery rate (FDR) < 0.05. GO and KEGG enrichment analyses of DEGs were conducted using the clusterProfiler package (p < 0.05). Key DEGs were selected for validation using quantitative real-time PCR (qPCR).

### 2.5 Quantitative real-time PCR validation

Reverse transcription and qRT-PCR were performed following the methods described by Liu et al. (2022) (Yonggang et al., 2023). Primer sequences used for transcript quantification are listed in **Supplementary Table 1**.

### 2.6 Statistical analysis

All experiments were conducted with at least three independent biological replicates. Data are expressed as mean ± standard deviation (SD). Statistical significance was analyzed using one-way analysis of variance (ANOVA) followed by Duncan’s multiple range test to compare differences among multiple treatment groups. For pairwise comparisons between JA-treated and control groups, Student’s t-test was employed. A *p*-value < 0.05 was considered statistically significant. Data visualization was performed using GraphPad Prism 9.0.

## 3 Results

### 3.1 Changes in phenotypic and physiological parameters under different temperature treatments

**Figure 1:**
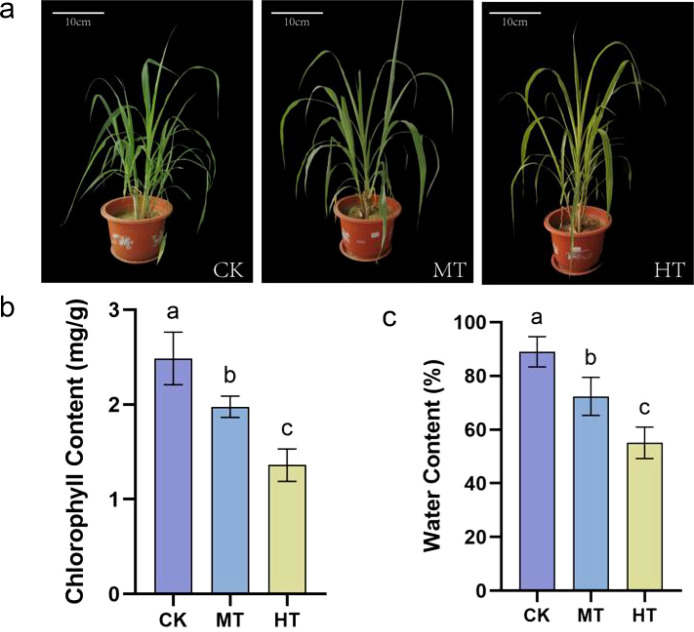
Phenotypic and physiological characterization of hybrid Pennisetum under different daytime temperature regimes. **(a)** Representative photographs of plants subjected to Control (CK, 28 °C/25 °C), Medium Temperature (MT, 37 °C/25 °C), and High Temperature (HT, 45 °C/25 °C) treatments for 5 days. Scale bars = 10 cm. **(b)** Quantification of leaf chlorophyll content and **(c)** leaf water content assessed after the 5-day exposure. Data are presented as mean ± SD. Different lowercase letters above the bars indicate statistically significant differences at *P* < 0.05, as determined by one-way ANOVA followed by Duncan’s multiple comparison test.

As shown in **Figure 1**, after 5 days of exposure to different temperature treatments, significant effects were observed on the leaf morphology and physiological characteristics of hybrid Pennisetum. Plants in the control group (CK) exhibited vigorous growth with bright green leaves, while those under moderate-temperature (MT) conditions showed slight leaf yellowing. In contrast, plants exposed to high-temperature (HT) conditions displayed pronounced chlorosis and wilting (**Figure 1a**).

Increasing temperature markedly inhibited chlorophyll accumulation and reduced leaf water content. As illustrated in **Figure 1b**, chlorophyll content was highest in the CK group, followed by the MT group, and was lowest in the HT group, showing a clear decreasing trend with rising temperature. Similarly, leaf water content (**Figure 1c**) also declined progressively across treatments, with the highest values in CK and the lowest in HT.

Collectively, these results indicate that heat stress significantly reduced chlorophyll and water contents in hybrid Pennisetum leaves, thereby exerting adverse effects on plant physiological activity and leaf health.

### 3.2 Transcriptomic and JA metabolism analysis of hybrid Pennisetum under heat stress

**Figure 2:**
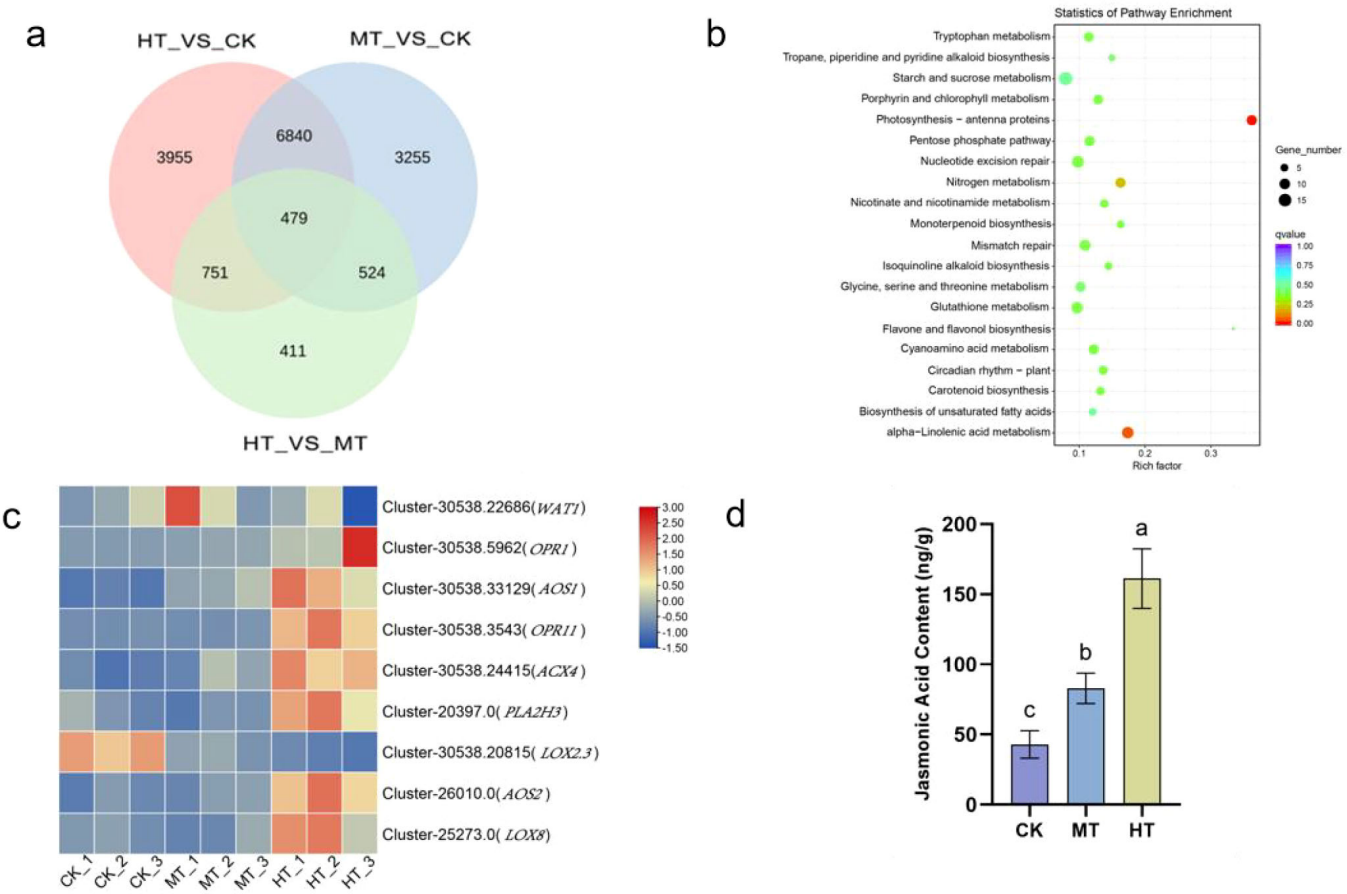
Transcriptomic and JA metabolism analysis of hybrid Pennisetum under different temperatures. **(a)** Venn diagram showing DEGs among CK, MT, and HT conditions. **(b)** KEGG enrichment analysis for MT vs. HT, highlighting significant enrichment in the α-linolenic acid metabolism pathway. **(c)** Heatmap of key genes in this pathway across CK, MT, and HT; expression is Z-score normalized. The color scale ranges from blue (low expression) to red (high expression). **(d)** JA content (ng/g FW) under different temperature conditions. Data are presented as mean ± SD. Statistical significance was determined using one-way ANOVA followed by Duncan’s multiple range test, as described in **Figure 1**; different letters indicate significant differences (*P* < 0.05). High temperature strongly activated α-linolenic acid metabolism and increased JA accumulation.

In order to elucidate the molecular mechanisms underlying the heat stress response in hybrid Pennisetum, RNA-seq analysis was performed on leaf samples collected from plants subjected to CK, MT, and HT conditions. Differentially expressed gene (DEG) analysis revealed substantial transcriptional reprogramming among the treatments (**Figure 2a**). Specifically, over 10,000 DEGs were identified in each comparison (HT vs. CK, MT vs. CK, and HT vs. MT), with 479 genes commonly expressed across all comparisons, indicating that heat stress triggered extensive gene expression regulation.

KEGG pathway enrichment analysis showed that the DEGs were predominantly associated with lipid metabolism and signal transduction pathways, including “α-linolenic acid metabolism,” “biosynthesis of unsaturated fatty acids,” and “glutamate metabolism.” Among these, the “α-linolenic acid metabolism” pathway, which is closely related to JA biosynthesis, was significantly enriched (**Figure 2b**), a finding suggesting that the JA biosynthetic pathway may play a pivotal role in the heat stress response of hybrid Pennisetum (Fatma et al., 2021).

Heatmap analysis of genes enriched in the α-linolenic acid metabolism pathway revealed that the expression levels of *OPR1*, *AOS1*, *OPR11*, *ACX4*, *PLA2H3*, *AOS2*, and *LOX8* were markedly upregulated with increasing temperature, whereas *LOX2* and *LOX3* exhibited downward trends; in contrast, *WAT1* showed no significant response to temperature variation (**Figure 2c**),*ACX4* encodes a peroxisomal acyl-CoA oxidase involved in the β-oxidation steps required for the conversion of OPDA to JA, whereas *WAT1* encodes a tonoplast-localized transporter previously associated with auxin homeostasis and secondary cell wall formation.

Finally, the JA contents in leaves under different temperature treatments are presented in **Figure 2d**. The results showed that JA accumulation increased significantly under the HT condition, reaching approximately 130 ng/g FW, compared with 90 ng/g FW under MT and 50 ng/g FW under CK. Different letters (a, b, c) indicate significant differences among treatments (P < 0.05); these findings suggest that heat stress promotes JA accumulation, which may contribute to the enhancement of thermotolerance in hybrid Pennisetum.

### 3.3 Phenotypic and physiological changes in hybrid Pennisetum under different JA concentrations and temperature treatments

**Figure 3:**
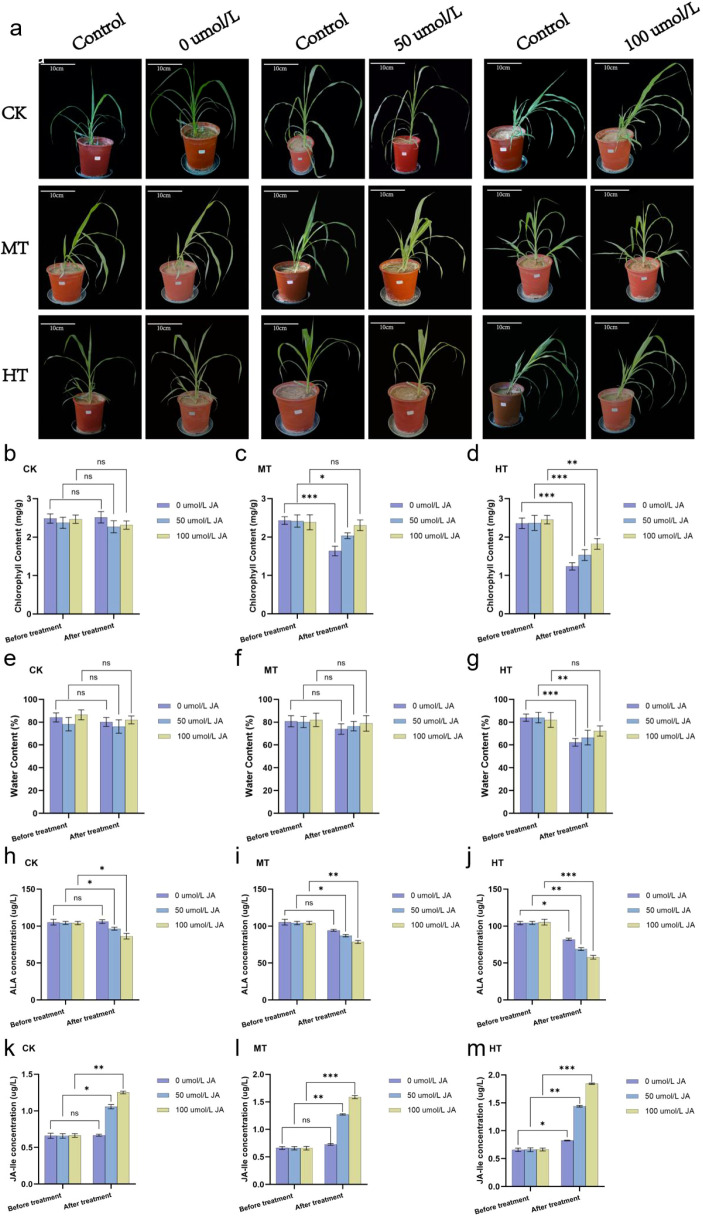
Effects of exogenous jasmonic acid (JA) concentrations and temperature treatments on the growth and physiological indicators of hybrid Pennisetum. **(a)** Leaf phenotypes of hybrid Pennisetum under different JA concentrations (0, 50, and 100 μmol/L) and temperature regimes [CK (28 °C), MT (35 °C), and HT (45 °C)]. Images in the “Control” column represent the initial state before stress, while other columns show phenotypes after 7 days of treatment; scale bars = 10 cm. **(b–d)** Total chlorophyll content (mg/g), **(e–g)** leaf relative water content (%), (h–j) ALA concentration (μg/L), and **(k–m)** JA-Ile concentration (μg/L) measured at two stages: “Before treatment” (Day 0) and “After treatment” (Day 7). Physiological parameters were evaluated under **(b, e, h, k)** CK, **(c, f, i, l)** MT, and **(d, g, j, m)** HT conditions. For each treatment, samples were collected from five independent plants, using the third fully expanded leaf from the apex for post-treatment analysis to ensure consistency. Brackets indicate statistical comparisons between JA-treated groups (50 or 100 μmol/L) and the untreated control (0 μmol/L) within the “After treatment” stage. Data are expressed as mean ± SD (n = 3). Statistical significance was assessed via one-way ANOVA followed by Duncan’s multiple range test: * *P* < 0.05, ** *P* < 0.01, *** *P* < 0.001; “ns” indicates no significant difference.

As shown in **Figure 3**, foliar application of different concentrations of JA under varying temperature conditions had significant impacts on the leaf morphology and physiological characteristics of hybrid Pennisetum.

Regarding leaf phenotypes (**Figure 3a**), plants grown under control temperature conditions (CK) maintained healthy green leaves across all treatments. Under moderate heat stress (MT), plants without JA application (0 μmol/L JA) exhibited slight leaf chlorosis, while those under high heat stress (HT) displayed severe yellowing and wilting, indicating evident heat-induced damage. With increasing JA concentration, the degree of chlorosis was progressively alleviated. Spraying 50 μmol/L JA markedly mitigated leaf yellowing under both MT and HT conditions, and, at 100 μmol/L JA, leaf greenness was further restored—particularly under HT—suggesting that exogenous JA effectively alleviates heat-induced leaf senescence and chlorophyll degradation.

The effects of JA treatment on chlorophyll content are presented in **Figures 3b–d**. At all temperature levels, plants treated with 50 μmol/L or 100 μmol/L of JA exhibited significantly higher chlorophyll content compared with untreated controls (0 μmol/L JA). Notably, under HT conditions, the decline in chlorophyll content was substantially reduced with the 100 μmol/L JA treatment, indicating that exogenous JA helps maintain chlorophyll stability and delays leaf senescence under heat stress.

A similar trend was observed for leaf relative water content (RWC) (**Figures 3e–g**). As temperature increased, RWC decreased across CK, MT, and HT conditions; however, JA-treated plants consistently maintained higher RWC values than untreated controls (P < 0.05). Among all treatments, plants treated with 100 μmol/L JA under HT conditions exhibited the highest RWC, suggesting that JA significantly enhances water retention capacity in leaves exposed to heat stress.

Furthermore, the application of exogenous JA significantly altered the accumulation of key stress-related metabolites (**Figures 3h–m**). For ALA, a vital precursor in the JA biosynthesis pathway, its concentration exhibited a distinct dose-dependent decline following JA application across all temperature regimes (**Figures 3h–j**). This decrease was most pronounced in plants treated with 100 μmol/L JA under HT conditions, suggesting that exogenous JA supplementation may accelerate the downstream metabolic conversion of internal ALA. Conversely, the concentration of JA-Ile, the active form of jasmonate signaling, showed a significant increase in response to JA spraying (**Figures 3k–m**). Under all temperature conditions, plants treated with 100 μmol/L JA displayed the highest JA-Ile levels compared to the 0 μmol/L JA controls, with the maximum accumulation observed under severe HT. This indicates that the exogenously applied JA is efficiently converted into the bioactive JA-Ile, potentially amplifying the internal stress-responsive signaling network.

Collectively, these results indicate that exogenous JA application effectively mitigates the detrimental effects of heat stress on hybrid Pennisetum by maintaining higher chlorophyll content and leaf water status, as well as modulating key endogenous jasmonate metabolites, thereby improving physiological performance and enhancing thermotolerance.

### 3.4 High-temperature stress induces activation of the α-linolenic acid metabolism pathway and enhances JA biosynthesis in hybrid Pennisetum

**Figure 4:**
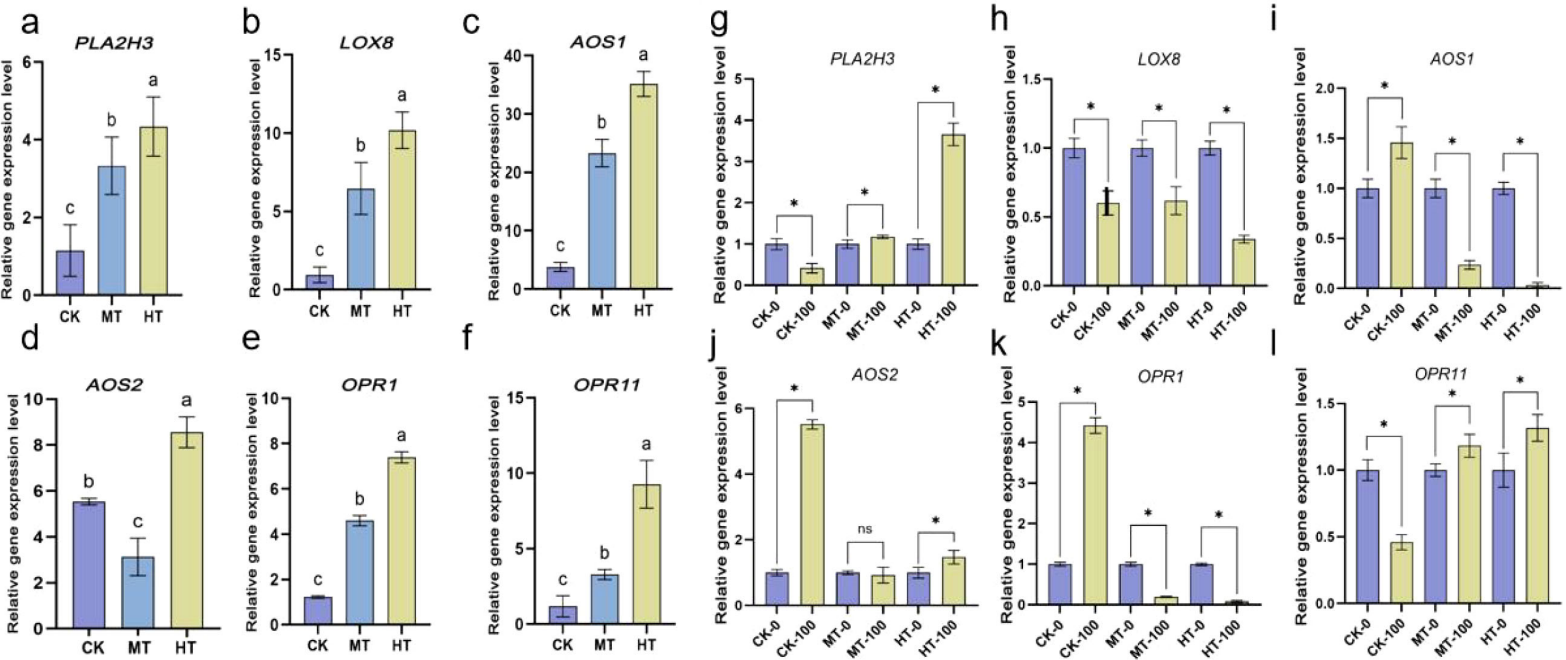
Relative expression levels of key genes in the α-linolenic acid and JA metabolic pathways of hybrid Pennisetum under different treatments. **(a–f)** qPCR analysis of six α-linolenic acid metabolism genes (PLA2H3, *LOX8*, *AOS1*, *AOS2*, *OPR1*, and *OPR11*) under CK, MT, and HT conditions. **(g–l)** Expression levels of the above genes with JA treatments (0 or 100 μmol/L) under CK, MT, and HT conditions. All leaf samples were collected after 7 days of continuous treatment with 0 μmol/L or 100 μmol/L JA under the specified temperature regimes. Relative quantification of gene expression was determined using the 2 ^−ΔΔCt^ method, normalized against the internal reference gene PpActin. For **(a–f)**, statistical significance among temperature treatments was assessed by one-way analysis of variance (ANOVA) followed by Duncan’s multiple range test, where different lowercase letters denote significant differences at *P* < 0.05. For **(g–l)**, the significance of differences between the 100 μmol/L JA treatment and its respective 0 μmol/L control at each temperature level was evaluated using Student’s t-test. Asterisks represent significant differences (*P* < 0.05); “ns” indicates no significance.

In order to verify the reliability of the RNA-seq results and further examine the expression dynamics of JA biosynthesis-related genes under heat stress, six key genes involved in the α-linolenic acid metabolism pathway—*PLA2H3*, *LOX8*, *AOS1*, *AOS2*, *OPR1*, and *OPR11*—were analyzed using quantitative real-time PCR (qPCR) (**Figures 4a–f**). The results revealed significant differences in expression levels of all six genes across temperature treatments.

As shown in **Figures 4a–f**,Under optimal temperature conditions (CK), the expression levels of the above genes remained relatively low. Under moderate heat stress (MT), expression levels were generally upregulated, and under severe heat stress (HT), all genes showed markedly elevated expression, reaching their highest levels. Among them, *AOS1*, *AOS2*, and *LOX8* exhibited the most pronounced inductions, increasing approximately 20–30 fold compared with CK, indicating that these genes are highly responsive to heat-induced signaling; similarly, *PLA2H3*, *OPR1*, and *OPR11* displayed consistent upregulation trends. Overall, the strong induction of key enzyme genes in the α-linolenic acid metabolism pathway was consistent with the RNA-seq enrichment analysis, confirming that heat stress activates the JA biosynthetic pathway in hybrid Pennisetum.

In order to further investigate the regulatory role of exogenous JA under different temperature conditions, qPCR was performed to examine the expression levels of the six key genes (*PLA2H3*, *LOX8*, *AOS1*, *AOS2*, *OPR1*, and *OPR11*) in response to JA application (**Figures 4g–l**); the results showed significant differences in expression levels across both temperature and JA treatments.

In plants without JA application (0 μmol/L JA), the expression levels of all six genes increased significantly with rising temperature; notably, *LOX8*, *AOS1*, and *OPR11* exhibited the highest expressions under HT, suggesting their crucial involvement in the heat-induced JA biosynthetic pathway.

“Additionally, a medium concentration treatment of 50 μmol/L JA was included (see **Supplementary Figure 1**). The results revealed that the transcriptional response of JA-related genes to varying concentrations was complex and gene-specific. While several genes displayed enhanced expression with increasing JA concentrations, others exhibited a non-linear response pattern; for instance, at the 50 μmol/L concentration, certain genes showed expression levels lower than those in the 0 or 100 μmol/L treatments, suggesting a more intricate regulatory mechanism or feedback inhibition at intermediate hormone levels.

Following exogenous JA application (100 μmol/L JA), the transcriptional response of the key biosynthetic genes displayed significant variation. Notably, *PLA2H3* exhibited a prominent and significant induction under high-temperature (HT) conditions compared to untreated controls (*P* < 0.05), suggesting that exogenous JA primarily enhances the initiation of the α-linolenic acid metabolism pathway by promoting the hydrolysis of membrane lipids. In contrast, genes involved in the downstream steps of the pathway, such as *LOX8* and *OPR11*, showed relatively limited responsiveness or even slight downward trends across different temperature regimes at the 7-day sampling time point. These results indicate that JA-mediated thermotolerance in hybrid Pennisetum may be primarily driven by the early-stage activation of the α-linolenic acid metabolism pathway through *PLA2H3* rather than the continuous upregulation of all downstream biosynthetic genes.

Taken together, exogenous JA treatment and heat stress displayed a clear synergistic effect, jointly promoting α-linolenic acid metabolism pathway activation and enhancing the expressions of JA biosynthetic genes, thereby improving thermotolerance in hybrid Pennisetum. High-temperature stress significantly induced activation of the α-linolenic acid metabolism pathway and increased endogenous JA accumulation, while exogenous JA further amplified the transcriptional responses of key genes within this pathway, forming a positive regulatory loop. The above findings indicate that JA mediates α-linolenic acid metabolism pathway activation and plays a critical molecular regulatory role in the development of heat tolerance in hybrid Pennisetum.

### 3.5 JA-mediated model of heat stress response in hybrid Pennisetum

**Figure 5:**
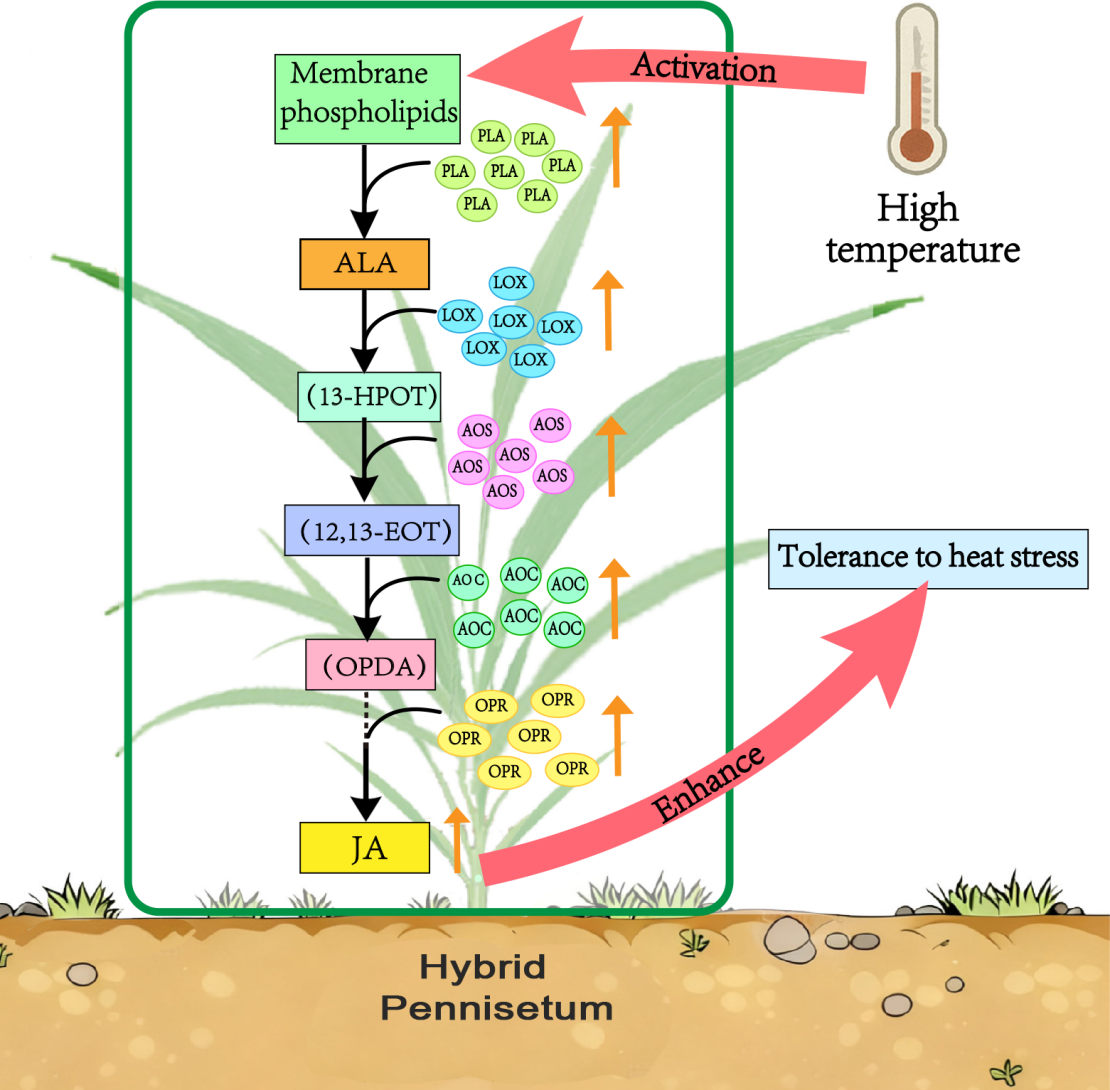
Proposed model for heat-induced JA biosynthesis and thermotolerance in hybrid Pennisetum. Under high-temperature stress, the Jasmonic acid (JA) biosynthetic pathway is activated through the upregulation of key enzymes. The process initiates with the hydrolysis of membrane phospholipids by phospholipase A (PLA) to release α-linolenic acid (ALA). ALA is sequentially converted into 13-HPOT by lipoxygenase (LOX), 12,13-EOT by allene oxide synthase (AOS), and cyclized to OPDA by allene oxide cyclase (AOC). Subsequently, OPDA is reduced to JA via 12-oxophytodienoic acid reductase (OPR). The resulting accumulation of JA triggers downstream defense responses, thereby enhancing thermotolerance in hybrid Pennisetum.

Based on the integrated results of transcriptomic sequencing and physiological–biochemical analyses, a regulatory model of the JA biosynthetic pathway in hybrid Pennisetum under heat stress was constructed (**Figure 5**). The findings indicate that high-temperature stress markedly induces the upregulation of phospholipase (PLA)-related genes, which promotes the degradation of membrane phospholipids and the release of α-linolenic acid (ALA), providing substrates for JA biosynthesis. Subsequently, the activities and transcript levels of several key rate-limiting enzymes, particularly lipoxygenase (LOX), allene oxide synthase (AOS), allene oxide cyclase (AOC), and 12-oxo-phytodienoic acid reductase (OPR), were significantly enhanced, driving the sequential conversion of ALA into intermediates such as 13-HPOT, 12,13-EOT, and cis-OPDA, ultimately leading to the accumulation of bioactive JA.

JA accumulation not only increased significantly during heat stress, but also activated the expressions of downstream defense-related genes, including antioxidant enzymes, heat shock proteins (HSPs), and osmoprotectant biosynthetic enzymes; this upregulation strengthened cell membrane stability and reactive oxygen species (ROS) scavenging capacity, thereby improving the thermotolerance of hybrid Pennisetum (Zhao et al., 2017; Sehar et al., 2023).

Collectively, this model integrates multi-omics and physiological evidence to systematically elucidate the activation pattern of the JA signaling pathway and its central role in the heat stress response of hybrid Pennisetum. The above results provide a theoretical framework for understanding the molecular mechanisms underlying heat adaptation in C4 grasses, offering new insights for improving thermotolerance in forage and ornamental species.

## 4 Discussion

### 4.1 Heat stress causes physiological damage to hybrid Pennisetum leaves

Chlorophyll content and leaf relative water content (RWC) are key physiological indicators reflecting photosynthetic efficiency and water balance status in plants (Tasir et al., 2018). In this study (**Figure 1**), as temperature increased, hybrid Pennisetum exhibited pronounced leaf chlorosis and dehydration, accompanied by significant declines in chlorophyll II content and RWC. The above observations indicate that heat stress disrupts photosynthetic function and cellular homeostasis in hybrid Pennisetum.

Similar findings have been reported in other species, such as rice, maize, and tall fescue, in which elevated temperature damages the photosystem II (PSII) reaction center, accelerates reactive oxygen species (ROS) accumulation, and triggers oxidative stress responses, ultimately leading to premature leaf senescence and decreased photosynthetic capacity (Foyer and Hanke, 2022; Meiling et al., 2023).

### 4.2 Heat stress induces activation of the α-linolenic acid metabolic pathway and promotes JA accumulation

RNA-seq analysis revealed that the α-linolenic acid metabolic pathway was significantly enriched in hybrid Pennisetum leaves under heat stress (**Figure 2**), accompanied by marked upregulation of several key genes involved in JA biosynthesis, particularly *PLA2H3*, *LOX8*, *AOS1*, *AOS2*, *OPR1*, and *OPR11*; this pathway represents the central route of JA synthesis, in which elevated temperature enhances the release and oxidation of α-linolenic acid from membrane lipids, thereby promoting endogenous JA biosynthesis. Notably, in this study, the *PLA2H3* gene, which encodes phospholipase A2, exhibited a remarkably strong response under heat stress. As the initiating enzyme of the ALA metabolic pathway, the activation of *PLA2H3* directly promotes the release of ALA from membrane lipids. Considering that ALA is not only a substrate for JA biosynthesis but also a key physiological signal for plants to perceive heat stress and regulate membrane fluidity, the heat-induced upregulation of *PLA2H3* provides the material foundation for the burst of JA synthesis and marks the initiation of early heat-defense through the remodeling of lipid metabolism in hybrid Pennisetum.

Similar phenomena have been observed in Arabidopsis thaliana (Guo et al., 2024) and Oryza sativa (Fatma et al., 2021), in which heat stress rapidly activates the transcription of JA biosynthetic genes, leading to elevated JA levels and subsequent activation of the heat stress response (HSR) pathway (Sharma et al., 2023).

Consistent with the transcriptomic data, JA quantification (**Figure 2d**) showed a significant increase in JA content under heat stress, confirming that JA plays a crucial role in heat signal perception and response in hybrid Pennisetum. Previous studies have demonstrated that JA not only participates in regulating the ROS-scavenging system, but also enhances membrane stability and photosystem heat tolerance by modulating membrane lipid fluidity and chlorophyll degradation pathways (Khan et al., 2025).

### 4.3 Exogenous JA alleviates heat-induced physiological damage

After foliar application of different JA concentrations (**Figure 3**), the degrees of leaf chlorosis in hybrid Pennisetum under moderate- and high-temperature stress were markedly reduced. Meanwhile, chlorophyll and leaf water contents significantly increased and showed positive correlations with JA concentration; these results indicate that exogenous JA effectively mitigates the physiological damage caused by heat stress.

Similar findings have been reported in cotton (Yang et al., 2020) and wheat (Rahman et al., 2024), in which JA enhances heat tolerance by increasing the activities of antioxidant enzymes (SOD, POD, and CAT), reducing ROS accumulation and promoting the synthesis of osmoprotectants, such as proline and soluble sugars (Zamora et al., 2021).

Crucially, our metabolic data reveal the internal dynamics of the jasmonate biosynthesis pathway in response to exogenous JA. We observed a significant decrease in ALA—the initial chloroplast-derived precursor for JA biosynthesis—alongside a striking, dose-dependent increase in JA-Ile. The reduction in ALA suggests that exogenous JA application may modulate endogenous lipid metabolism, potentially accelerating the downstream metabolic flux or exerting feedback regulation on upstream precursors. More importantly, JA-Ile is the authentic bioactive signaling molecule that binds to the COI1 receptor to activate JA-responsive gene expression (Wasternack and Strnad, 2016). The massive accumulation of JA-Ile, which peaked under HT conditions with 100 μmol/L JA treatment, demonstrates that exogenously applied JA is efficiently absorbed and conjugated into its active form. This heat-enhanced conjugation likely serves as a rapid response strategy to strongly trigger the stress-signaling cascade.

The mechanism by which JA enhances plant thermotolerance lies not only in the self-amplification of its signaling but also in its broad regulation of downstream executors. Although antioxidant enzyme activities were not directly measured in this study, the significant maintenance of chlorophyll content and the alleviation of phenotypic damage following exogenous JA treatment indirectly demonstrate that JA likely clears heat-induced reactive oxygen species (ROS) by activating the antioxidant defense system (e.g., SOD, POD, and CAT). Secondary mining of the transcriptomic data corroborates this, showing that JA-induced differentially expressed genes are significantly enriched in pathways related to photosystem protection and redox homeostasis maintenance. This suggests that JA acts as a signaling hub, resisting oxidative damage caused by extreme temperatures by stabilizing core proteins of the PSII reaction center and enhancing cellular scavenging capacity.

In addition, JA may help maintain leaf water balance by regulating stomatal aperture and controlling transpiration rates (Wasternack and Hause, 2013), which could explain the significant increase in leaf water content observed in JA-treated plants in this study.

### 4.4 Synergistic effects and positive feedback regulation between exogenous JA and heat stress

qPCR validation further revealed the complex interaction between exogenous JA and heat stress. The study found that *PLA2H3* exhibited the most significant induction under the combined treatment of heat and exogenous JA, supporting the hypothesis that JA can reinforce the initiation of the ALA metabolic flux through positive feedback. However, we observed that downstream genes such as *LOX8* and *OPR11* displayed a complex, non-linear response pattern to exogenous JA, with their expression levels not showing continuous fold increases after 7 days of treatment and even decreasing at certain concentrations. This phenomenon likely reflects the signaling homeostasis within the plant; once endogenous signals accumulate to a certain threshold, the plant suppresses the transcription of downstream biosynthetic enzymes via negative feedback to avoid the energetic burden of an excessive stress response.

### 4.5 Significance and application prospects

Comprehensive analysis indicates that hybrid Pennisetum enhances its heat tolerance by activating the α-linolenic acid metabolism and JA signaling pathways, thereby regulating gene expression and physiological metabolism to maintain cellular homeostasis under heat stress. Exogenous JA application further amplifies this process, improving the physiological status and stress resistance of the plants (Kazan and Manners, 2008).

Although it remains challenging to implement genetic transformation in the non-model hybrid Pennisetum to provide direct causal evidence, the “exogenous JA rescue experiment” conducted in this study offers compelling physiological evidence: supplementing JA significantly reversed the physiological collapse caused by extreme heat. This phenomenon demonstrates that JA is not a byproduct of heat stress but a decisive regulator in the heat-defense network. Future research will focus on utilizing proteomics to further elucidate the direct interactions between JA-perceiving proteins (e.g., JAZs) and heat shock proteins, thereby constructing a more complete regulatory map (Wasternack and Strnad, 2016).

The above mechanism not only elucidates the molecular basis of heat tolerance in hybrid Pennisetum, but also provides a theoretical framework and practical direction for enhancing heat resistance in turfgrasses and forage crops through the manipulation of the JA signaling pathway (Liu et al., 2022; Liu et al., 2024).

## Data Availability

The data presented in the study have been deposited in the NCBI repository under BioProject accession number PRJNA1467233. The corresponding SRA accession numbers are SRR38669818–SRR38669826, and the BioSample accession numbers are SAMN60211623–SAMN60211631.
