# Supplementary material for: Jasmonic acid enhances thermotolerance in hybrid Pennisetum via activation of the α-linolenic acid metabolism pathway
Source: Front Plant Sci. 2026 May 26;17:1756886. doi: 10.3389/fpls.2026.1756886 (PMC13246406; doi:10.3389/fpls.2026.1756886)
Supplement: Supplementary file 1 [file DataSheet1.docx]

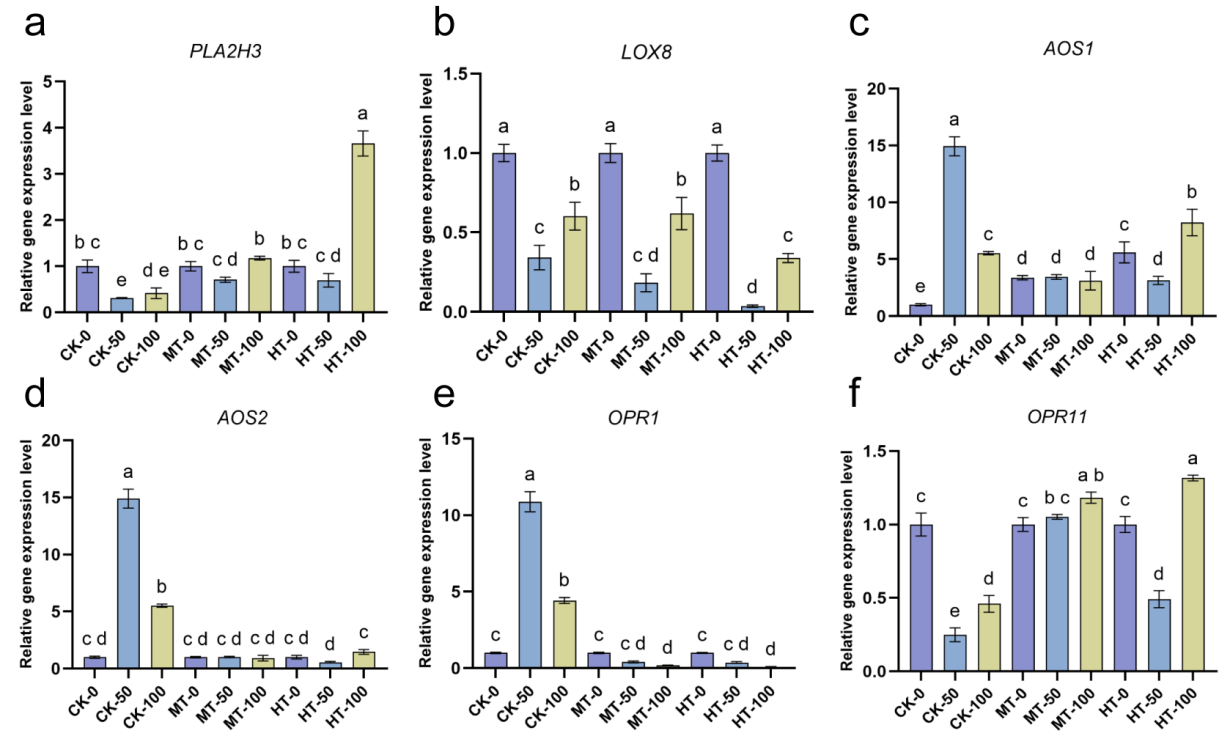


**Figure S1. Relative expression levels of JA biosynthetic genes under combined temperature and exogenous JA treatments.**(a-f)The transcript abundance of six key genes involved in the JA biosynthesis pathway (*PLA2H3*, *LOX8*, *AOS1*, *AOS2*, *OPR1*, and *OPR11*) was analyzed using qRT-PCR. Plants were subjected to three temperature regimes (CK, MT, and HT) and treated with varying concentrations of exogenous JA (0, 50, and 100 μmol/L). Data are presented as means ± standard deviation (SD) of three biological replicates (n = 3). Different lowercase letters above the bars indicate statistically significant differences among treatments as determined by Duncan’s multiple range test (p < 0.05).
